# Situating implementation science (IS) in res(IS)tance: a conceptual frame toward the integration of scholarship from the black radical tradition

**DOI:** 10.3389/fpubh.2023.1286156

**Published:** 2024-01-11

**Authors:** Cory D. Bradley, Whitney C. Irie, Elvin H. Geng

**Affiliations:** ^1^Center for Dissemination and Implementation in the Institute for Public Health at Washington University School of Medicine in St. Louis, St. Louis, MO, United States; ^2^School of Social Work, Boston College, Chestnut Hill, MA, United States

**Keywords:** health equity, social justice, implementation science, structural racism, resistance, black studies, public health, health disparities

## Abstract

This manuscript undertakes a disciplinary self-critique of the field of implementation science, a field which attempts to bridge the gap between evidence-based interventions and their practical application. Despite the heightened emphasis on health equity and racial disparities, the field’s current discourse is limited by key epistemic shortcomings. First, even though prevalence of implementation gaps between racialized groups in the United States necessitates a comprehensive understanding of the systems perpetuating these disparities, the field does not operate with a general explanation for disparities not as a failure of systems, but a system historically and structural designed to produce disparities. Second, the field has attempted to address disparities without adequate dialog with a broad tradition of anti-racist and anti-colonial sociology, history and epistemology, and therefore risks a decontextualized analysis of disparities and under-informed approaches to achieving equity. Fortunately, scholarship from the Black radical tradition (BRT), such as the Public Health Critical Race Praxis (PHCRP), Critical Race Theory (CRT), and more broadly conceptual frameworks from post-modern, anti-colonial, Black feminist studies and social epistemology can offer to implementation science frameworks that center power dynamics and racialized oppression. This epistemic re-alignment of implementation research to “center at the margins” can enable the field of implementation science to more critically examine and dismantle systems that perpetuate racial inequalities in access to and utilization of health interventions. For example, normalization and dynamic fit, which are thought to be key mechanisms of implementation, are revealed in the light of this tradition of scholarship to be potentially problematic acquiescence to oppressive systems. Drawing from the concept of resistance anchored in the scholarship of the Black radical tradition as well as contemporary social epistemology such as the work of José Medina and Maria Fricker about epistemic justice, the authors further advance that implementation science could make more substantial contributions to the dismantling of racialized systems and actively work toward health justice through the transdisciplinary lens of resistance. This is a call to action for integrating implementation science with critical philosophical and theoretical perspectives rooted in Black studies and related insights, which have been acquired through the struggle for social justice, to inform the design of implementation strategies and research projects that improve health services and health outcomes for health disparity populations.

## 1 Current lens: lifting the veil of implementation science

### 1.1 Implementation science: the “gap” and its discontents

The emergent prioritization of equity in implementation science demands, first and foremost, a disciplinary self-critique to ensure that the field’s perspectives are sufficiently rooted in equity-making concepts. At this point, the motivating narrative for the field of implementation science is thought to be well understood. Implementation science begins with recognizing a wealth of technologies—evidence-based interventions—have been produced that can improve health and well-being. The field’s unifying analysis is the recognition that these scientific products have not been adequately used in ‘real world’ settings. Often cited is the finding that Americans receive about half of indicated health interventions (1). Many examples of basic, affordable interventions (such as beta-blockers after heart attacks) took decades to become routine (2). Hence, the science of implementation has generated a focus toward the development and empirical testing of implementation strategies as methods to improve the adoption and sustained utilization of interventions in diverse contexts.

*“Resistance is a choice made in community, made possible by community and informed by memory, tradition and witness…Resistance is our heritage. And resistance is our healing.”*

*—***Robin D. G. Kelley (3), p. 161**

While this offers a compelling account of a crisis in the use of evidence-based interventions, the field has not widely provided unifying or even middle-range explanations or theories for racialized disparities in the gap between research evidence and the routine use of that evidence in practice. An examination of the implementation of evidence-based practices in the United States, however, reveals not only the fact that many interventions are sub-optimally used but another unavoidable and equally obvious feature: gaps in the use or uptake of evidence-based interventions differ across racialized groups. In fact, evidence for interventions that could reduce disparities and improve population health is limited because too few interventions have successfully been disseminated and translated into diverse practice settings, which Cooper et al. characterize as “an implementation of evidence gap” (4). Moreover, racism and discrimination have been identified as a fundamental cause by which those racialized evidence gaps are performed (5–7). For example, in one study, change in condom use for Black participants was effective and increased over time for Black participants when residential segregation was relatively low and the attitudes of White people toward Black people were relatively favorable. Moreover, the study found that tailoring of interventions for targeted participants improved intervention success by mitigating the statistical effect of White people’s racist attitudes toward Black people (8). Racialized differences appear in virtually all health conditions and span diverse delivery modalities (e.g., preventative, acute, behavioral or surgical). This “implementation of evidence gap” means that our society and science have failed to fully deliver the promises^1^ of translational science benefits, and this chasm can be characterized as inequity—avoidable, unnecessary, and unjust differences in health status (10). While it is true that there are failures of implementation in privileged or dominant groups too, there are not many examples of implementation gaps and health outcomes that are not differential by race in the United States. Racialized health disparities, therefore, cannot be considered a “special case of implementation failure” (11), but rather—in the terminology of Critical Race Theory (CRT)—an ordinary and routine feature of implementing systems. Following the field’s raison d’être, the prevalence of racialized health gaps in implementation implies that the entirety of implementation science must be committed to understanding and undoing the work of systems that maintain *racialized* gaps in health outcomes.

Given the centrality of disparities, it is surprising that theories and frameworks most frequently invoked in implementation science are relatively silent on this issue. Diffusion of Innovations theory, which is widely cited and considered an influential forerunner of implementation science, viewed the spread of innovations as occurring in a social system over time, the kinetics which could be determined by characteristics of the social system or characteristics of the intervention (e.g., trialability) (12). But Rogers did not discuss racialized social systems that exclude and disadvantage particular individuals and, therefore, is silent on the role of racism in spread and access, whether passive or managed. More recently, the popularized Consolidated Framework for Implementation Research (CFIR) offers a compilation of domains useful for identifying several potential determinants of implementation success, but it does not privilege any particular set of relationships of power (13). If looking for an explanation of racial disparities in implementation, beyond suggesting relevant domains of inquiry, CFIR is limited in promoting critical race-consciousness regarding the ubiquity of racialized differences in the use of evidence-based intervention (14). Recent calls for an emphasis on equity in implementation have given this topic long overdue prominence (15–17). To do so, implementation science as a field must be intentional about incorporating perspectives that help us understand not only that such barriers exist but *why* those gaps in adoption, implementation, sustainability, and scale-up of evidence-based interventions and practices are often racially differential. Understanding *how* they come into being so that they may be undone. Furthermore, we must remain uncomfortable with why our explanations to date have been so quiet on an issue that is so clearly a historical and contemporary hallmark of the formation of American society.

### 1.2 The science of systems-breaking and justice-making

Paul Batalen’s insight that “every system is perfectly designed to deliver the results it delivers (p. 1059–1,061)” (18) prompts a crucial question when examining the prevalence of racialized health disparities: could these gaps be the intended outcome of systems and processes that were designed to reproduce those gaps? Systems, after all, are designed to hold things in place—to maintain the status quo. This notion aligns with the systems justification theory, asserting that individuals are inclined to justify and defend existing social, economic, and political systems. According to this theory, people find satisfaction in the status quo as a means to alleviate existential anxiety stemming from uncertainty, threat and social discord. Moreover, experiments have suggested that exposure to system criticism or threat can actually increase commitment to the status quo as a system-justifying response (19).

This leads to the intriguing proposition that inequities perceived as a “special case of implementation failure” might be the result of systems—both implicitly and explicitly—crafted to disproportionately concentrate access to and utility of goods and services, including evidence-based health interventions, innovations, and technologies. Such a reinterpretation of evidence-to-practice gaps holds profound implications, not only for enhancing the capacity of implementation research to address racialized disparities but also for shaping the field’s identity and response to the societal challenges contributing to these implementation failures and inequitable health outcomes.

First, seeing racialized implementation gaps as a product of the design of social stratification presents an opportunity to align implementation research with the intellectual and philosophical traditions of critical social theories explaining power and oppression such as postmodern and anti-colonial scholarship, Black and ethnic studies including Critical Race Theory (CRT) and the Public Health Critical Race Praxis (PHCRP) which is a framework derived from empirical methods in CRT, Black Feminisms and Black Queer Theory alongside the poignant and prolific scholarship of scholar-activists such as Frantz Fanon, W.E.B. Dubois, and Ida B. Wells and countless more contemporary scholars whose work has centered the struggle for racial justice. In short, implementation research has the potential to enrich its insights by integrating critical perspectives that have long focused on the dynamics of power and racialized oppression within American institutions and systems. By doing so, it enables exploration of the impact of social, legal and broader systems, extending beyond the realm of health systems, that contribute to the persistence of health inequalities. That is particularly useful in examining structural racism as it has been identified as a system, in collusion with capitalism and other structures of oppression that produces a distribution of goods and services which disadvantages non-white populations (20–26).

Rachel Hardeman and J’Mag Karbeah critique health services research as consisting of methods that are “fundamentally flawed because they rarely identify, name, and interrogate the influence of white supremacy, the white racial frame, and structural racism (25) (p.770).” Robin D. G. Kelley, in his essay entitled *Black Study, Black Struggle* advocates that “struggle, deep study, and critique [offered through Black studies]” gets us to “the root—the historical, political, social, cultural, ideological, material, economic root—of oppression” in order to unearth and generate pathways to liberation. Inherent to such scholarly frameworks, he adds, is the task of illuminating the hidden, as “most structures of oppression and all of their various entanglements are simply not visible and not felt (3), p. 164.” Understanding racialized implementation gaps must draw guidance from frameworks, models, and theories that center on understanding racialized hierarchies of power. Inattentiveness to the normative, sometimes silent influences of racialized socialization on social real-world contexts affects scientific theorizing and the credibility of the claims science makes (27).

Much is at stake in this (re)alignment. Implementation gaps investigated without such frameworks will not yield implementation strategies that adequately and accurately counteract systems of oppression. Health delivery systems and their processes of implementation require reconfiguration, and reconstruction, akin to the kinds of non-reformist reforms that have been pursued to achieve political, economic, and social transformation strategies and maneuvers (26). To be concrete, the notion of “fitting” interventions into systems is considered a good thing in implementation, and feasibility is championed. Yet fit into racialized systems and structures may merely perpetuate racialized differences in access and “feasibility” within unmodified racialized systems implies conformity to those systems.

What we as a field come to believe to be true depends on social relations, social structures and attendant assumptions that are hidden but ultimately could perpetuate harm in practice, policies, and subsequent implementation strategies. Systems and norms reproduce racialized inequities when those factors are not explicitly named, interrogated, and disrupted. Thus, implementation research needs an orientation that allows us to detect and respond to social forces that exert their influence through mechanisms of context, both materially and nonmaterially, that structure the delivery, receipt, and operationalization of health interventions and research evidence. With implementation science poised to act as a tool for justice-making, its aspiration should be to resist the unjust influences of the worlds we make and that make us, to eliminate those barriers and deploy local assets and strategies to move us forward along the path of health justice. Throughout this paper, we seek to characterize the resistance that is required.

### 1.3 The “work” racism does upon implementation context

Context can sometimes be treated as a problem in the social sciences and, more specifically, implementation science (28). However, a scientific understanding of context is fundamental to understanding what works for whom and under what conditions. Understanding context facilitates a deeper engagement as to why some interventions or practices fail to be embedded or are difficult to de-normalize or de-implement so that new approaches can be embedded. One analysis noted in 2004, “Investigation of how intervention effects are modified by context is a new methodological frontier in community intervention trial research (pp. 788) (29).” Still today, it is widely acknowledged that the implementation and effectiveness of evidence-based interventions are inextricably linked to the dynamic and multilevel contexts in which they are implemented (30). Yet although implicated in racial health disparities and implementation outcomes such as reach, adoption, adaptation, and sustainability, for example, *methods* for considering the dynamic interplay between health interventions/innovations and their implementation in racialized context have not been well articulated; nor have the mechanisms by which racialized social systems affect outcomes of implementation adequately explained within the field.

Unquestionably, attention to racial socialization in implementation is significant to context. Pfadenhauer et al. define the function of context beyond the role of backdrop for the implementation of the intervention; rather, context possesses an active and dynamic role exerted through interaction, influence, modification, facilitation, and constraint upon the intervention and its implementation (31). Through their definition, then it must also be apparent that racialized contexts wield an impact upon implementation. Further characterizing the work context enacts upon implementation, May notes that ‘contexts’ are not so much “organizational” or static as organiz*ing* in non-linear, emergent, and dynamic ways. As a point of leverage, Hawe and colleagues suggest that because there is an interaction between interventions and their implementation, there exists the potential for system transformation whereby “the evolution of new structures of interaction and new shared meanings” emerges (32) to enhance the intervention’s context fit, a necessary condition for successful implementation. Rather than implementation science taking a pose that ignores or controls for context, its task can be actively seeking to intervene by transforming the context in which health innovations are deployed to enhance equitable uptake and embedding (33); therein, opportunities within implementation scholarship to assess and adapt racialized contexts to facilitate successful implementation figures as a critical tool of resistance (34).

### 1.4 Resisting normalization: understanding racialized implementation failures through denormalization

It is a foregone conclusion that the implementation gap is racialized. Such racialization is perpetuated by a system of rules and regulations that are both explicit and implied. Eduardo Bonilla-Silva defines racialization as “the extension of racial meanings to actors and practices (21), p.515.” The everyday phenomenon of race-making in tandem with various concrete, material practices (e.g., policies, distribution of determinants of health) ultimately manifests the practices, policies, and mental models that produce advantages for some and disadvantages for others (35). Disparities in the uptake, implementation, sustainability, and scale-up of evidence-based interventions undergird population health disparities, which differ by race across all age groups, conditions, and geographies in the US. From Batalen’s notion of a system’s intended design, these resulting failures of implementation arguably are successes at maintaining the American caste^2^ through racialized social systems: that is, they are doing what they were intended to do (18). Juxtaposing the impact of racism as a public health crisis (38) on implementation failure means that a central task for implementation research is identifying and understanding how such social pathologies in our systems operate across myriad phases and activities of implementation. Furthermore, it gestures toward the actions that should be taken to counteract this. The stakes are high – implementation science can only solve the problems it identifies. Otherwise, misidentifying the problem and why those problems exist might mean our efforts at solutions will also miss the mark. As Kimberley Crenshaw has stated of Critical Race Theory (CRT), an intellectual and activist framework fostered through the Black radical tradition as an analytic tool to systematically detect and analyze racial inequality in the US legal framework, CRT enables one to see the problem of racial domination more clearly; otherwise, she remarks, “If you cannot see a problem, you cannot solve it (39).” Implementation science needs a lens to see the problem of racism (40).

Alas, it is not, however, a forgone conclusion that the field of public health and implementation science adeptly perceives the mechanisms of racism and the impact of racialized social systems as central to driving implementation gaps; consequently, these fields risk (re)producing the epistemically numbing qualities that can unknowingly perpetuate the normalization of work that sustains racial inequities. Vital to the elimination of health inequities, Hardeman and Karbeah admonish (25):

“…health services researchers must emancipate ourselves from the dominant white supremacist framing that has touched every aspect of our science. We must strive to make what for so long has been invisible in health services research visible –there are lives depending on it (p.779).”

A resistance approach seeks to do just that. Toward the mobilization of cognitive activism (41), resistance reflects movements in research inquiry that interrogate, disrupt, and counter hegemonic and taken-for-granted assumptions long established. Those assumptions tend to uphold partial, singular, and excluding narratives while *othering* and delegitimizing perspectives deemed outside of that frame as inferior. In response, resistance consciousness counters through the transdisciplinary use of philosophical and critical theoretical tools to uncover less visible relations among knowledge and regimes of power in a given context, system, research design, or implementation practice. Most significantly, resistance approaches tend to be identifiable among marginalized knowers through their various forms of communicative expressions, yet go unacknowledged by mainstream perspectives which results in the exclusion of those knowledges as irrelevant and inferior to the perspective of the dominant group. Throughout the rest of this paper, we forge the conceptualization of a resistance approach (interchangeable with resistance framework, resistance consciousness, resistance lens) for implementation science by motivating philosophical and critical theoretical tools to make the role of racialization and racial bias more visible in implementation research and practice, and ultimately counteracted through strategies designed to promote social justice.

## 2 Different lens: elaborating a resistance approach through an integration of social epistemology and BRT scholarship

### 2.1 The inherent necessity of a resistance lens in implementation science

In accordance with CRT, Bonilla-Silva underscores that participation in systemic racism is “normative and routinized,” yet “if systems have continuous productive and reproductive force, then [necessarily] resistance becomes a unifying approach to disrupt these systems (21).” The resistance framework strategically seeks to undermine unjust or oppressive hegemonic social meanings and power relations; examining implementation failures through the interactional nature of resistance focuses the central role of power in implementation and efforts to attain health equity. Thus, we place resistance at the center of implementation science’s response to racialized oppression in three senses. First, and foremost because there is something *wrong* that must be resisted to *right*. According to Anderson’s account toward a philosophical approach to justice, theorizing toward change must begin from an account of the wrong: systematic disadvantages that have been imposed (42). A just approach to the translation and implementation of evidence-based findings into communities, therefore, must begin with an account of the harm of racialized social systems that enduringly exist in the present rather than a presumption of justice and fairness referred to as post-racialism or color-blindness. Resistant consciousness that does not grasp for the ideal should enable an analysis that reveals the presuppositions of our most habitually expressed behaviors and open the analysis to a set of alternative assumptions that bring into existence methods and strategies that counter the status quo and potentially promote racial justice and health equity (43).

Second, is that scholarship about implementation must resist the otherwise unseen normalization of racialized inequities. The philosophy of social epistemology and Black radical scholarship invoke theorizing that recognizes that “the normalization of a presumed justice and the concomitant *ab*normalization of injustice have ideological effects which contribute to the invisibility of everyday injustices as well as the formation of active bodies of ignorance that perpetuate the injustices and desensitize us to the suffering they cause (Medina, p.13).” Initiating implementation inquiry from a resistance consciousness situates an analysis of implementation failure to resist explanations that bury the implications of racial oppression and rather facilitate the identification, classification, and design of implementation strategies that will disrupt the underlying factors of systemic racism in the context in which implementation failure occurs.

Third, without a resistance consciousness rooted in BRT which assists to detect those underlying realities, we are involuntarily and, at times, willfully complicit in the normalization of racialized inequities. Bonilla-Silva’s theory of racialized social systems contends that the bulk of white [people’s] participation in systemic racism is “normative and routinized^3^,” necessarily making resistance an active and deliberate rather than passive effort (p. 524). Thus, CRT surfaces the sources of racialized disparities by posing, “How is racism working here?” in order to unearth racialized perspectives, ideologies, and artifacts perhaps normalized beyond conscious awareness. Such an analysis destabilizes the insidious influence of latent racism in social systems through an explicit appreciation and interrogation directed at exploring how implementation processes are shaped. CRT, daughter of the legacies of scholarship within BRT, potentially serves as a tool of resistance in the analysis of implementation context by identifying the work that is done and work that should be undone which manifests in collective practices, mechanisms and strategies that reproduce racial domination.

### 2.2 Critical race theory as exemplar of resistance

Attention to a structural understanding of disparities may be newly heightened for implementation science. Still, it has long been the focus of scholarship from the Black radical tradition (BRT), including anti-colonial, antiracist, and Black feminist traditions (e.g., Sojourner Truth’s ‘Ain’t I A Woman’, The Combahee River Collective). Fortunate for implementation research consequently, BRT offers several insights through a diversity of communicative engagements (such as cultural esthetics of art, poetry and music, speeches, historical narratives, intellectual and activist collectives, accounts of social movements and civil rights engagements, etc.) to draw on and apply in health disparities research. Instead of depending on solutions for racialized disparities exclusively in the archival disciplinary knowledge of implementation science literature, we should first recognize that the absence of these frameworks or related constructs in implementation science is in and of itself a symptom of color-blindness and therefore complicit, and part of the problem (45). However, implementation science could remedy this limitation by earnestly studying anti-racist scholarship outside of the implementation and health sciences canons. Pursuing multi-disciplinarity in dissemination and implementation investigations, especially that which is specific to the conditions that sustain racialized disparities by utilizing BRT scholarship, potentially disrupts the hegemony of scientific knowledge and its harmful (re)production rooted in the philosophy of science approaches that dictate the conduct of scientific inquiry. To address the influence of colonization and imperialism, philosopher Lewis Gordon points to emanant insights from BRT articulated by Frantz Fanon, a Francophone Afro-Caribbean psychiatrist, philosopher, and activist against coloniality who argued that addressing colonization at the level of method requires a suspension of method: a “method of no method” as Lewis Gordon describes:

“…as he [Frantz Fanon] argued in the first chapter of *Black Skin, White Masks*, colonization is also manifested by its means of implementation. Such instruments are also epistemological, and if the disciplinary practices that construct the modern colonized subject as subhuman are to be interrogated, that includes, as well, the presuppositions of unprejudiced interrogation (46), p.89.”

While there are many to draw on, extending core tenants of CRT as a theoretical lens and approach to anti-racist implementation science uniquely offers a resistance-enabling path of inquiry. CRT proliferates a range of analytic observations that ultimately recast the problem of racism not so much as rooted in individual malice but positioned within the structures of systems (47). The tenets, methodological approaches, and strategies of CRT were organized into a framework by a group of legal scholars of color in the 1980s, yet CRT has migrated across many disciplines, including education, psychology, cultural studies, public health, political science, and philosophy (47). CRT defines a set of anti-racist tenets and methodological approaches to knowledge production through transdisciplinary scholarship, which guide subsequent strategies for action. CRT tenets, methodological approaches, and claims are enmeshed with the rich intellectual and activist legacy of the Black Radical Tradition.

CRT arrived in the discourse of justice because of a perceived inadequacy of the prevailing definition of racism framed by liberal civil rights discourse, which largely defined racism as “discrete, easily identifiable, invariably intentional, always irrational acts that are perpetrated by bad actors” (47). CRT contests this definition, attending to the fact that racism is not always manifested in discrete actions; rather, it can also be the result of the intersections and actions of multiple institutions of society. Thus, because they are not always specific behaviors, they can manifest unaware and unintentionally (47). The potential for CRT to advance health equity in health services research is that it proposes to make visible the relationship between power and social roles and the patterns and habits that make up racialized hierarchies of domination, which are often invisible. Supporting our paper’s characterization of a resistance approach, Foucault admonishes that “in order to understand how resistance works,” we need to understand “the strictly relational character of power relations” including more relevantly to this discussion, the relational character of hierarchical racialized power relations among the intervention and implementation assemblages which include the implementing team as well as the setting or context (48).

The implications of a CRT analysis are considerable: what if our justice-making institutions are fundamentally unjust, it asks. While legal scholars have contended with that question for decades, public health imminently confronts a similar inquiry: what if mechanisms for creating population health improvements, influencing the adoption/uptake and implementation and sustainability of health interventions, fundamentally produce and reproduce inequities in health outcomes? Or at the least harbor remnants and shards of the injurious, pervasive, and historical patterning of racism and social exclusion? If that is accepted as true, then it problematizes a public health and implementation scholarship that goes uncontested; absent of centering an interrogation of discriminatory systems, the task of implementation concedes to what has been inherited. While the insights offered through CRT hold that racial subordination is structured in social relations, the methods that empirically substantiate this in the social sciences have not been as well developed. The historical and theoretical critiques that are platformed by CRT analysis do not “offer a measurable basis” *per se* but contribute to the integration of critical race perspectives by prompting resistance to “theoretical and methodological silos” that preclude a deeper understanding of race and racism (20, 49, 50).

An explanation as to one mechanism by which CRT enables resistance in scientific analysis can be inferred through Medina’s conceptualization of epistemic resistance: that is, it operates by disrupting the status quo through an interrogation of silent and taken-for-granted institutional rules and regimes. He notes, “Critical Race Theory teach[es] us the importance of unmasking and undoing the process of the social construction of our perspective, of interrupting the flow of familiarity and obviousness, making the familiar unfamiliar and the obvious bizarre (42), p. 19.” This process, Medina advocates, ultimately cultivates a “self-estrangement” and openness to perplexity in which epistemic resistance is enshrined and manifested (42).

A robust translation of CRT in public health and health services research is exemplified by Drs. Ford & Airhihenbuwa in the Public Health Critical Race praxis (PHCRP), which is intended to guide health equity researchers and practitioners to explain and challenge power hierarchies that undergird health inequities (51, 52). The PHCRP is the first public health framework that translates CRT for empirical research. It was developed in response to the disconnect between public health frameworks that identify racism as a social determinant of health and racial theorizations that are critical to identifying, understanding, and addressing racism as the perpetrator of racialized disparities. PHCRP advances health equity and social justice with the intent of dismantling public health practices, systems, and structures that produce racialized inequities through multiple methods, including “counter-storytelling.” Counter-storytelling is inherently resistant and a foundational method of CRT. Another example, among many, of PHCRP resistance orientation is through its principled inquiry anchored in the ‘ordinariness of racism’, a principle which sensitizes and enable’s one’s perceptions of logics that perpetuate the normality of racism.

### 2.3 A metaphor to unfold the process of deploying resistance through CRT inquiry

Camara Jones elaborates on a metaphor first introduced by Beverly Daniel Tatum in her book *Why Are All The Black Kids Sitting in the Cafeteria and Other Conversations About Race* which we will use to motivate the transformative process of resistance and principles of anti-racist action involved in resistance to racism (53). In the metaphor, racism is characterized as a conveyor belt moving people to and through racism as they go about living their ordinary lives. Upon realizing the conveyor belt is moving toward racism, people can respond by turning their back to move against that direction in order to chart a different path than the conveyor belt is taking everyone. As one walks in the opposite direction, Jones highlights the first opportunity for anti-racist action occurs as one moves in the opposite direction of racism though inconvenienced by running into and bumping into people moving in the direction toward racism and its outcomes: that is to actively name racism among individuals moving with the flow or status quo. As people seek to resist the belt’s movement toward racism, they seek to dismantle the conveyor belt’s motor—the source of the racialized power; this engages the second principle of anti-racism as resistant action, which requires asking and seeking to understand: “how is racism operating here?” Upon understanding how racism operates and identifying its lever(s), the opportunity arises to engage the third principle of active antiracist resistance, which Jones describes as organizing and strategizing to act with others to dismantle the system (conveyor belt motor) and put in place a system (engine/motor) that leads to the development of one’s fullest health potential. The conveyer belt metaphor, or the contention that racism is both ordinary and ubiquitous moving us all along, at times stealthily, illustrates the core tenant necessitating a resistant implementation science.

Power gains force and traction through social relational structures, yet those structures are dynamic, much like the conveyor belt, and comprise or structure the process as much as they are made by the process. Eduardo Bonilla-Silva, articulating systemic racism, argues that we all participate in the reproduction of the racialized order through behaviors and actions that are normatively structured such that none is outside of systemic racism, thus supporting the argument that participation in systemic racism is “normative and routinized,” which requires resistance while also making resistance even more of a challenge (54). Opportunities for intervention must be analyzed and strategized with intentionality to attend to those nuances. Such normative participation in racialized structures in everyday life reproduces racial order beyond patently “racist” actions and behaviors. Though individual race-related behavior may vary, the “path of least resistance” reproduces the racial status quo in ways that extend beyond overt racially motivated behavior. From the racialized social systems perspective, the key to confronting systemic racism is identifying the collective practices, mechanisms, and behaviors that reproduce racial domination. Within the normalization process theory’s (NPT) capture of implementation (55), the key analytic is “characterizing the work that is done” to reproduce racial domination. Identifying that work requires an analysis of the normative and often unconscious behavior, actions, or negligence to act (which is referred to as epistemic ignorance) rooted in hegemonic systems. Uncovering and dislodging them requires resistance to the status quo.

The language of resistance points to possibilities for alternative approaches to implementation outside the frame of present realities largely responsible for the reproduction of racialized hierarchies by inviting action through strategies of implementation that are designed to promote racial justice. These possibilities invite emancipatory and liberatory ways of knowing and doing that eschew oppressive forces of power and generate political action toward health equity.

In the framework of resistance, CRT, for example, can support implementation processes to advance health equity by focusing on the ways that racism co-constitutes social context as well as encourages action to counter racist contexts by embedding justice-oriented approaches and principles through strategies of dissemination and implementation. A failing of public health research and practice is its “tendency to treat racism as…an easily identifiable and treatable hazard that individuals can be taught to avoid.” However, as Chandra Ford notes, “It is more appropriate to consider racism an integral element of the social context in which all populations exist and within which all studies of health disparities are conducted (31), p. 481.” CRT holds that racism is not something that will disappear rather, it mandates ongoing, iterative work on that problem. As such, an analysis advancing equitable implementation of health interventions should be processually iterative and facilitate normalizing a practice and way of doing and working that can be monitored and analyzed through continual engagement and relentless inquiry concerning racism and racialized perspectives, which is embodied in CRT and well-translated through the PHCRP tenets.

### 2.4 Resistance combats epistemic injustice and overcomes the injustice of epistemic resilience

Jose Medina in his text, *The Epistemology of Resistance: Gender & Racial Oppression, Epistemic Injustice, and Resistant Imaginations* (42) offers a socially and politically philosophical perspective on resistance that conjoins scholarship from the Black radical tradition. He deploys a concept called ‘epistemic resistance’ which is defined as “the use of epistemic resources (knowledge production and knowledge translation capacities and abilities) to undermine and change oppressive normative structures and the complacent cognitive-affective functioning that sustains those structures...(Medina, p.3).” For our purposes, epistemic resources in implementation research include tools such as the development of research questions, research designs, data collection, analytic methods, use of theories/frameworks/models, participatory implementation approaches, implementation methods and strategies such as the guidance by ERIC (Expert Recommendations for Implementing Change) as well as dissemination and implementation practice.

When forms of epistemic ignorance (i.e., research perspectives that are silent about racism) are operational and the norm, the consequence are forms of epistemic injustice. Features of racialized social systems potentially enact epistemic injustice on implementation research and practice through several paths. One of those paths particularly relevant to knowledge/evidence translation which occurs in implementation research impacts on processes and activities of knowing that can result in what is known as epistemic injustice. Epistemic injustice is a concept thought to be introduced as early as the mid to late 1800s by Black female intellectuals and activists such as Anna Julia Cooper though the expression has been coined and popularized by philosopher Maria Fricker (56). Epistemic injustice describes a wrong done to someone in their capacity as a knower (57) and manifests in the exclusion of marginalized and oppressed people from being heard and understood by others in communicative expressions. It manifests in exclusion from the following:

1. being heard and understood by others in the diverse expressions of communication. That is, prejudice is enacted by the receiver of knowledge predicated upon the identity of the knower which serves to undermine the credibility of the knowledge holder resulting in a dysfunctional and incomplete knowledge dissemination. This form of epistemic injustice Fricker calls testimonial injustice (57);
2. contributing to social understandings and interpretations of the lived experiences and understandings about marginalized and oppressed people. That involves, at the systemic level, the identity-based exclusion of groups of knowers from participating in the shaping of collective understandings regarding their humanity; it is an expression of epistemic injustice Fricker identifies as hermeneutical injustice.
3. inequitable participation in debate, discussion, inquiry, and offering ideas for consideration toward posing alternative possibilities (58). It involves the discriminatory mistreatment of the epistemic agency of members of marginalized groups wherein the exercise of that agency is “unfairly constrained, manipulated, or subverted (42, 57, 58).

Epistemic injustice, occurring in different forms and functions as described, is viciously maintained and sustained by access to boundless resources scoured through colonialist and appropriative means. As such, efforts to transform structures and systems of oppression are often thwarted by their innate ability to reform and rebound; this is referred to as epistemic resilience and is further defined by Rogers as “the phenomenon whereby an epistemological system remains stable despite counterevidence or attempts to alter it” (59). It is from here where resistance calls upon us through the ephemeral voice of Audre Lorde to venture beyond the “master’s tools” (60), which includes the dominant, hegemonic epistemological systems and resources of oppression and to utilize tools that effectively dismantle epistemic injustice using resources designed, defined, and determined toward catalyzing transformation through resistance. Rogers highlights resistance as the antidote to this unavoidable form of epistemic resilience—a resistance appropriately birthed outside the bounds of dominant epistemology and destined to unearth its statute. Hence, we advocate for resistance as a tool for justice-making and a critical tenet of an equitable, anti-racist implementation science. In other words, to advance implementation science for equity and dismantle systems of oppression fortified by epistemic resilience, we should call upon resistance through the scholarship of the BRT. For resistance is an epistemically just response.

## 3 Designing resistant strategies for implementation

“Design is the process by which the politics of one world become the constraints on another.”—Fred Turner (61).

### 3.1 Resistance and the design of multi-faceted and multi-level implementation strategies

All design reproduces ways of being, knowing, and doing, which is referred to as the ontological dimension of design (62). To concede that every system delivers what it was “designed for” means that when racial health disparities result from a failure of equitable implementation, we must soberly consider that the design of health interventions, including the design of strategies for their translation, implementation, and sustainment, have the capacity to silently reproduce systemic racial injustices to the being, knowing and doing of minoritized and marginalized populations which we observe in those self-same disparate health outcomes. As astutely insighted by Mohamed, Png & Isaac in their discussion of the challenge of deploying decolonial theory in artificial intelligence (AI):

“…whereas in a previous era, the intention to deepen racial inequities was more explicit, today *coded* inequity is perpetuated precisely because those who design and adopt such tools are not thinking carefully about systemic racism” (63).

Plainly, Mohamed et al. warn that the tools of ‘new’ health innovations, interventions, technologies, and practices recapitulate and perpetuate racial injustice and harm in contemporary racialized social systems in the absence of apt consideration as to the influence of systemic racism. Similarly to AI, the charge remains for justice-making in the science of implementation to discover how to break, disrupt, and decipher the “coded inequity.” Ontological design then would suggest that the inclusion of equitable implementation efforts and access to optimal health in their design has the capacity to affirmatively impact the ways of being, knowing, and doing of populations.

Racialized social systems enact their effect on implementation through the design and deployment of implementation strategies, generating designs rooted in assumptions that can be violent and hostile to individuals who have been “Othered” by those systems; they may widen inequities, or at best leave racialized barriers to equitable implementation in place, unmoved, and untransformed. It may also mean that assets or facilitators of implementation among marginalized communities and through indigenous knowledge are devalued and therefore ignored in the design of strategies, robbed of their capacity to generate equitable outcomes. Implementation strategies may necessarily need to look and be more disruptive to the status quo of operations when that way of doing “business as usual” could be holding inequity in place. Contrary to technocracy, implementation may need to take on a social justice and disruptive tone to mark significant change toward health equity.

Reframing the reasons for implementation gaps as “designed for” by racialized social systems and institutions means that the central task of implementation research is to identify and understand how certain drivers reproduce racialized gaps and disparities in outcomes and then to design strategies that counteract these processes through a resistant imagination. Rather than solutions that target people and their behavior as the problem, developing a resistant implementation strategy chiefly involves shifting the gaze by asking what kind of structural world (environment) must exist to produce equitable implementation and health outcomes, and then resistant strategy design goes about acting on that. Consequently, implementation strategies are obliged to secure not only the routinization and embedding of interventions in practice but also to do so in a way that is equitable and can potentially mitigate group differences in health outcomes. Racial disparities in health outcomes persist in part when the evidence of health interventions has limited reach or poor translation for identity groups disproportionately impacted by disadvantage; those poor dissemination outcomes persist without dissemination and implementation strategies that challenge the status quo which makes it acceptable (normative). Thus, it is critical that scholarship about implementation no longer ignore or be silent about racism; rather, brings it center stage to uncover and explore its performance in dynamic contexts while interacting with features of the intervention and selected implementation strategies.

Another path by which racialized social systems may critically enact their effect on implementation is through muting or disabling the necessary (re)arrangements of relational and material capacities, including power, that would facilitate successful implementation and embedding. Aligning implementation strategies with social justice unfolds the opportunity for health interventions and their implementation through resistance-informed multifaceted and multilevel impacts to counteract the existing structures of racism that have been normatively embedded in US social systems and institutions. In essence, implementation strategies can be made to become artifacts of resistance against the status quo and enable opportunities and capabilities that promote improved health outcomes. Learning from the strategies of social justice movements and social resistance efforts chronicled in the scholarship of the BRT could be penetrative, resonant and relevant for designing strategies and approaches that build community or collective power and raise value for the relational and material agency required for health improvements. As the complexity of racism and its impact on the health of Black Americans is better grasped, the more equipped the science will be to successfully intervene rather than applying strategies that cause harm because they are ill-informed (49) and misdirected.

### 3.2 Design justice, denormalization and disrupting coherence

If it is accepted that the system is designed to produce inequities, how can strategies to advance implementation at the same time resist the tendencies to implement inequitably? Are there particular implementation strategies that are more reflective of and sustain the function of systems rooted in racial hiearchies or racialized power? Is there an interrogation of the underpinning principles by which implementation strategies are generated or deployed? What is the relationship of those core underpinning principles to projects of emancipation, liberty, and social justice? Implementation typically takes the concept of “coherence” as the point of departure. Per the normalization process theory, coherence is a fundamental accomplishment or “generative mechanism” in normalization. Coherence involves an agent’s understanding of an intervention, in context, and its *possibilities* [italics authors] as antecedent to other generative mechanisms such as cognitive participation and collective action (50).

Resistant strategy design for implementation should begin envisioned by resistant imagination, disruptive meaning-making, and critical appraisal of the default collective imaginaries. Resistant implementation means that the interaction between what we do and the meanings it can take on starts with a shared recognition or coherence about the system as producing results that are as designed but unacceptable. In a sense, this could be considered “denormalization.” If the universe of possibilities as (re)imagined by actors is the basis of establishing coherence in order to reach normalization, we propose that the first act of resistance must be one of collective imagination to collectively amplify the possibilities and disrupt coherence for the former in order to achieve denormalization. The actors must imagine different possibilities to ensure that those take on meaning, leading to a particular kind of coherence. It can be characterized as coherence to a vision of justice and health equity and, at the same time, willing to be made uncomfortable, inconvenienced, and disrupted in order to achieve that vision, which requires a resistance consciousness. Coherence work is as integral to building practice as it is to changing it (50). The role of champions is perceivably significant when establishing collective coherence in implementation that interrogates and breaks with systems of oppression as well as is open to designing better futures.

Delving into the forefront of implementation research for equity involves the active process of unpacking, dissecting, and adapting strategies—an approach that diverges from perpetuating a closed, “one world” design ontology. When designing implementation strategies with a focus toward justice, Arturo Escobar’s perspective in *Designs for the Pluriverse* offers valuable guidance. Escobar advocates for a design orientation centered on collaborative and place-based practices that recognize the interconnectedness of all people, beings and materiality of the earth (62, 64). Similarly, the capture of a resistant imagination in the design of implementation strategies the same seems resonant with deploying a resistant imagination in the design of implementation strategies and methods suggests a need for collaboration, a grounded sense of place and an amplification of the interdependence among all beings and the environment. To counteract the unequal distribution of benefits and burdens inherent in design, a resistance-conscious approach to strategy design should prioritize the reorganization of processes around three key principles: (1) collective capabilities, assets, and capacity building, (2) collective liberation, and (3) ecological sustainability. This shift toward a more inclusive, interconnected and environmentally mindful framework is essential for fostering equitable outcomes and dismantling the structural barriers that perpetuate disparities (62, 65).

## 4 Conclusion: SANKOFA invoking the legacy and promise of black scholarship

Sankofa is a principle derived from the Akan people of Ghana; its direct translation in the Twi language is “to retrieve,” and its expanded translation is that “it is not taboo to fetch what is at risk of being left behind.” Sankofa is a reminder to identify what is being left behind during the sweeping momentum of scientific advancement. Instead of looking for tools and solutions to address racialized disparities exclusively in implementation science or even behavioral sciences, we should first recognize their absence or silence in related frameworks and related constructs in implementation science, which in and of itself is complicit and part of the problem. Though its theories, models, and frameworks have been critiqued for not addressing issues that are critical to advancing health equity, such as power, reflexivity, and inequality (66), implementation science *can* ameliorate such deficits through a resistance consciousness activated by an earnest study and inclusion of scholarship beyond the discipline that speaks to issues of power, equity, and social justice used to interrogate the hegemony that maintains inequality. Fortunately, for implementation science, there is an opportunity to seek, understand, and retrieve insights from the scholarship of Black studies broadly. The Black radical tradition is a disciplinary focus most notably recognized as Black studies or African American studies in higher education. In an interview with Robin D. G. Kelley, the Distinguished Professor and Gary B. Nash Endowed Chair in U.S. History and professor of African American studies at UCLA, Keeanga-Yamahatta Taylor, writing for the New Yorker, features his articulation of Black studies, with a paramount clarification that looms over the distortions of an anti-Black political agenda (67). Kelley says:

“…It [Black studies] is really about examining Black lives: the structures that produce premature death, that make us vulnerable; the ideas that both invent Blackness and render Black people less than human; and perhaps most important, the struggle to secure a different future.” He continues, “It’s about interrogating racial categories, understanding the persistence of inequality…and finally the way that African people really tried to remake and re-envision the world, through art, through ideas, through social movements, through literature, through study in action” (3).

As such it is our stance that the scientific translation of health innovations and technologies charged with the explicit goal of advancing health equity will be enhanced guided by the scholarly insights, analyses and insights emanating from scholarship within the BRT discourse. We offer the praxis of resistance which we analytically motivate through linking connotations of social and political philosophy as well as the critical scholarship rooted in the Black radical tradition with implementation science and efforts to pursue health equity.

Despite racialization or the practice of race-making serving as “the template for subordination and oppression” for multiple racial groups, it can also be redemptively a “template for resistance to many forms of marginalization and domination (68).” Omi & Winant in *Racial Formation in the United States* reflect that drawn upon myriad theoretical insights embodied within BRT, new social movements of The Black Movement in the 1960s and 1970s too were inspired by the strategies, organizing tactics, political demands, and more broadly challenges to practices of subordination and exclusion. This paper elaborates upon that claim by conceptualizing the relevance and significance of embodying a resistance consciousness in the pursuit of centering the elimination of racialized health disparities in the science and practice of the translation and implementation of health technologies and novel approaches and innovations to improve population health.

We champion the integration of resistance within implementation science, a stance aimed at actively challenging racial oppression and dismantling prevailing modes of knowledge that are embedded with racial bias. In drawing inspiration from Pierre Bourdieu who raises concerns about the potential drawbacks of an “established social science” impeding progressive and innovative research, we advocate with Guttormsen & David for the use of epistemic reflexivity to counteract this risk (69). Epistemic reflexivity involves an approach to science that aligns “not only with one’s scientific training but also against it”—a resonant call for epistemic resistance. To generate the essential friction required for this resistance, we propose engagement with scholarship of the Black radical tradition and critical social theories as powerful candidates. Such scholarship could serve as a catalyst for questioning established norms of racial hierarchy and their intersectionalities, fostering a dynamic environment that encourages critical reflection and innovative thinking within implementation science (69, 70).”

Finally, for the sake of solidarity it is important to acknowledge this analytic approach in no way isolates racism as the only contextual factor underlying racialized and other group health disparities to the exclusion of colluding oppressions (23). Noteworthy, the practice of “making up people” racially or otherwise has proven transferrable and has been imported to other marginalized identities and political struggles (68). Therefore, it is held that the latitude and range inherent within the resistance praxis of BRT allows for the transmutation of a resistance approach across studies involving multiple other oppressive ideological domains that belie inequality.

## Data availability statement

The original contributions presented in the study are included in the article/supplementary material, further inquiries can be directed to the corresponding author.

## Author contributions

CB: Conceptualization, Formal Analysis, Data Curation, Investigation, Methodology, Writing – original draft, Writing – review & editing. WI: Formal analysis, Writing – original draft, Writing – review & editing. EG: Conceptualization, Formal analysis, Investigation, Methodology, Writing – original draft, Writing – review & editing.

## Footnotes

^1^The discursive frame, “a failure to fully deliver on its promises,” hearkens to Dr. Martin Luther King Jr.’s I Have A Dream speech (9) in which he stated, “In a sense we have come to our Nation’s Capital to cash a check. When the architects of our great republic wrote the magnificent words of the Constitution and the Declaration of Independence, they were signing a promissory note to which every American was to fall heir...It is obvious today that America has defaulted on this promissory note insofar as her citizens of color are concerned. Instead of honoring this sacred obligation, America has given its colored people a bad check, a check that has come back marked “insufficient funds.” But we refuse to believe that the bank of justice is bankrupt. We refuse to believe that there are insufficient funds in the great vaults of opportunity of this nation. So we have come to cash this check, a check that will give us upon demand the riches of freedom and security of justice…Those who hope that the colored Americans needed to blow off steam and will now be content will have a rude awakening if the nation returns to business as usual.”

^2^From the viewpoint of Pulitzer prize-winning author Isabel Wilkerson in her book Caste: The Origins of Our Discontents (36), racism is an insufficient term for the systemic oppression Black people in America experience. She prefers the frame of America as having a “caste” system defined as an “artificial hierarchy that helps determine standing and respect, assumptions of beauty and competence and even who gets benefit of the doubt and access to resources.” The use of “caste” is intended to emphasize the infrastructure of racialized social systems that we often cannot see but undergirds the injustices and disparities we often do see (37).

^3^Bonilla-Silva’s perspective on “White people’s participation in systemic racism” is cited in this discussion to point to the effect of being socially racialized in whiteness or white privilege, which predominately occurs to/among individuals racially identified as White and is an inherent feature of the American racialized hierarchy. Liu has said, “Critiques of whiteness as a condition do not assume an essentialist character among people who are racialized as White” but is rather concerned with the structural dimensions of racial power (44). In fact, Eduardo Bonilla-Silva goes on to argue that we all participate in the reproduction of the racialized order through behaviors and actions that are normatively structured so that no one is truly outside of systemic racism.
